# Supplementary material for: Longitudinally tracking personal physiomes for precision management of childhood epilepsy
Source: PLOS Digit Health. 2022 Dec 19;1(12):e0000161. doi: 10.1371/journal.pdig.0000161 (PMC9931296; doi:10.1371/journal.pdig.0000161)
Supplement: S1 Text — (DOCX) [file pdig.0000161.s001.docx]

**Supporting Information**

1. **Patient recruitment guideline and overview**
2. **Data collection by wristband**
3. **Seizure identification**
4. **Head-to-head comparison between wristband and medical device on HR**
5. **PAWO pipeline**
6. **Physiome and activity study**
7. **Principal Component Analysis (PCA) on developmental physiologies during childhood development**
8. **Construct prediction model for seizure onset**
9. **Construction of the cloud-based prediction system leveraging the pre-trained model**

## Patient recruitment guideline and overview

| Total number of inpatient children with epilepsy considered during the study | 341 |
| --- | --- |
| Total number of inpatient children with epilepsy, consented to participate in the study | 127 |
| Total number of admitted patients in the study | 99 (66 in Discovery, 33 in Replication) |

**Inclusion criteria:**

Children age from 0~18 with focal epilepsy, generalized epilepsy and epileptic encephalopathy were included; epilepsy was diagnosed by experienced neurology physicians from Children’s Hospital of Zhejiang University, School of Medicine, according to 2017 International League Against Epilepsy (ILAE) classification of the epilepsies (doi: 10.1111/epi.13709) described as follows:

1. At least two unprovoked (or reflex) seizures occurring >24 h apart;
2. One unprovoked (or reflex) seizure and a probability of further seizures similar to the general recurrence risk (at least 60%) after two unprovoked seizures, occurring over the next 10 years
3. Diagnosis of an epilepsy syndrome

**Exclusion criteria:**

1. Non-epileptic seizures accompanying alterations in consciousness, sensation, motor function, and mentality were excluded, such as syncope, hysterics, transient ischemic attack, hypoglycemia, hypocalcemia, somnambulism, psychotic disorders, and extrapyramidal diseases, etc.
2. Participants withdrew due to various reasons during the study were considered excluded.

The final study cohort consisted of a total of 99 children. They were further divided into discovery (66) and replication (33) cohorts, respectively. The study was approved by the ZJUCH review board, and the parents of the children provided written informed consent. These children were all previously diagnosed with epilepsy, and they were admitted to the hospital upon requested by their parents and guardians due to recent seizure episodes. Each patient was admitted for v-EEG monitoring at the hospital. Each patient wore the Microsoft Band wristband at all times including during sleep; the wristband was not taken off during physical activities. During most of the time, the patients’ activities were not restricted while at the same time they were encouraged to rest.

## 2. Data collection by wristband

Two types of smart-bands were used in this study, Microsoft Band 1 and Band 2, which were equipped with a microphone, 3-axis accelerometer, 3-axis gyroscope (Microsoft Band 2 only), light sensor, thermometer, UV sensor, capacitance sensor, optical heart rate meter, GPS, skin electric response detector (Microsoft Band 2 only) and barometer sensors. Each wristband is connected via Bluetooth to a nearby smartphone, and the measurements are transmitted to the smartphone at the real time. This smartphone has a pre-installed dedicated App, which allows the caregiver to record it when the child is having a seizure or other unusual events. This smartphone is also connected to a remote central cloud data server via Wi-Fi or cellular network in real time. The recorded data is securely stored on the remote server for storage and analysis. The App has an intuitive user interface, which allows the user to access and display historical data, either recent data stored locally on the cell phone or remotely on the cloud server. In addition to the smartphone placed near the patient, the patient’s real time and historical data can also be accessed securely through the cloud server by any remote smart phone. The App and cloud server have security features to prevent hacking and data breach. The private data has been anonymized to remove identifiable personal information.

## 3. Seizure identification

Each child had been previously diagnosed of being epileptic prior to this study. While they were in the hospital, they were monitored around the clock by caregivers or nurses. Due to lack of EEG equipment, for the majority of the time, a patient was not subjected to EEG measurement; instead, the seizure episodes and other unusual events were observed by the caregivers and recorded by using the App on the smartphone.

## 4. Head-to-head comparison between smart-band and medical devices on Heart Rates (HR)

We compared Microsoft Band with an arm-worn medical grade device, OMRON HEM-6230, for its accuracy in measuring heart rate (HR). We chose one human subject (male, age 25, and in good health) for this comparison. We tested each device on this subject under both resting and active states for 30 minutes each. In addition, a trained observer recorded every read change from the arm-worn OMRON HEM-6230 device.

**Supplemental Figure S1A** compares the HR data acquired from the smart wristband (open blue circles) and from the medical device (red asterisks) in resting state, while **Figure S1B** compares the data in the active state. The measurements acquired by the smart wristband were smoothed by the hardware built-in algorithm; the medical device acquired HR data in every 30 seconds. We next calculated the absolute linearity of these two data series by using the following equation (**Equation 1**); the measurements from the medical device were deemed as gold standard.

|  | $L_{ab}=\frac{\Delta Y_{ab,max}}{Y_{ab,max}-Y_{ab,min}}\times100\%$ | (1) |
| --- | --- | --- |

**Supplemental Figure S1C** and **S1D** show the calculated absolute linearity results in the resting state and in the active state, respectively. The red line is the theoretical linearity measured by the medical device, the cyan dotted line was the theoretical linearity measured by the smart wristband, while the blue dash line is the best-fit line between these two data series. The calculated error rate was 0.4% and 1.2% for the resting and the active state, respectively.

## 5. PAWO pipeline

We designed PAWO (***Platform for Analyzing Wearable Output***) pipeline to organize and process sensor signals acquired from the wearable devices, which consisted of four coherent modules: *stream processing, pre-processing, signal analysis and multi-signals time alignment*. The table below outlines key technical details in the platform. The individual modules are described in detail below.

|  | **ACC** | **GYR** | **HR** | **GSR** |
| --- | --- | --- | --- | --- |
| **Frequency** | 8Hz | 8Hz | 1Hz | 0.1 Hz |
| **Preprocessing** | Yes | Yes | Yes | Yes |
| **Additional analyses** | Motion detection | - | HR/HRV analysis | - |
| **Time alignment across multi-signal channels** | Alignment based on time stamps of every second | | | |
| **Sliding windows** | Window for signal QC: non-overlapping window for every minute  Window for Motion detection: 80% overlap for every five minutes  Window for detecting abnormal HR: 80% overlap for every five minutes | | | |

**5.1 Stream processing module**

PAWO ensures efficient and robust data transmissions between the wristband and the smartphone, which is capable of handling large volume of data from diverse sensor types. We made the following modifications to the accompanied SDK/API toolkits: (1) we encoded sampling time as 32-bit Unix timestamp accuracy (achieving the resolution to millisecond), providing sufficient resolution for time alignment across events and among data from different sensor types. (2) We packaged sensor type identifiers with the time stamp as well as the acquired sensor readouts into individual data streams, which are subsequently transmitted to the connected mobile phones. (3) We also made the system automatically check the wearing/idle status of the devices, and this signal is separately transmitted to the connected mobile phones. (4) These signals are packaged and sorted automatically at the backend cloud sever.

**5.2 Pre-processing module**

The pre-processing module was designed for signal quality identification (SQI), denoising, signal smoothing, and adjustment for personal physiological base lines.

5.2.1 Signal Quality Identification (SQI). This module identifies the wearing status of the wristband and removes signals when a device was not active, e.g., during battery charging. This ensures the collection of valid and meaningful data in our study. SQI also filters missing data from the acquired sensor signals. Specifically, we used sliding window method to identify time segments with high rate of missing data. The sliding windows were 60 second in length and non-overlapping. The system removes time segments where more than 60% of the data were missing.

5.2.2 Signal Denoising***.*** We adopted discrete wavelet transformation to denoise non-stationary signals such as HR and ACC in this study. The process was divided into the following three steps. (1) Wavelet decomposition of the acquired signals. We implemented wavelet transformation and derived the high frequency coefficients associated with input signals that contained noise. (2) We then thresholded the high frequency coefficients. (3) Wavelet re-construction. We reconstructed the input signal by integrating the thresholded high-frequency coefficients and the originally derived low-frequency coefficients to achieve signal denoising. In our practice, we elected to use DB6 as the wavelet basis function, which has been widely used for processing physiological signals such as heart sound and ECG. We chose to decompose input signals at the level of five when analyzing ACC and GYR signals, where input signals could be decomposed into one low-frequency component and five high-frequency components. Because ACC and GYR had sampling frequencies of 8Hz, the frequency bandwidths of the first to the fifth high-frequency signals were set as D1:2hz ~ 4Hz, D2:1hz ~ 2Hz, D3: 0.5Hz ~ 1Hz, D4: 0.25Hz ~ 0.5Hz and D5: 0.1Hz ~ 0.25Hz. The approximate low-frequency bandwidth was 0 ~ 0.1Hz. The frequency ranges of the acceleration and angular velocity components is 0.3 ~ 3.5Hz for analyzing epilepsy activities (Poh et al 2012). Based on previous empirical estimate, the frequency ranges from the first to fourth detail layers are expected to be in the range of 0.25Hz ~ 4Hz, which should contain most of the energy of the input signal. Therefore, we directly set the fifth layer coefficient zero.

The HR signals were first converted into R-R interval (RRI) signal. The frequency distribution of RRI was generally concentrated in the ranges of ultra-low frequency (< 0.04hz), low frequency (0.04-0.15hz) and high-frequency bands (0.15-0.4hz). The bandwidth of high frequency signal components was set as D1: 0.25hz ~ 0.5Hz, D2: 0.125hz ~ 0.25hz, D3: 0.06hz ~ 0.125hz, D3: 0.03hz ~ 0.06hz. The bandwidth of the low frequency approximation layer was 0 ~ 0.03Hz. The frequency range of layer 1 to layer 4 was 0.03hz ~ 0.5Hz., which mostly covered the analysis of HRV from low frequencies to high frequencies.

When thresholding high-frequency components, we selected the hard threshold operator as the threshold function:

|  | $\hat{\omega}_{j,k}=\left\{ \begin{matrix} \omega_{j,k}, & \left\vert\omega_{j,k} \right\vert\geq\mu\\ 0, & \left\vert\omega_{j,k} \right\vert<\mu\end{matrix} \right.$ | (2) |
| --- | --- | --- |

The hard threshold can overcome computing challenges such as singular points in input signals. For seizure detection, singular point signals might contain clinically relevant information. The commonly used VisuShrink method was used to select the threshold.

For signal reconstruction, we adopted the Mallat algorithm to decompose input signals, and designed low-pass and high-pass filters to derive the approximate low-frequency and high-frequency coefficients. After thresholding the high-frequency components, we performed step-wise signal reconstruction as follows:

|  | $x\left( n \right)=\sum kA_{j,k}\tilde{H}\left( k-2n \right)+\sum_{j=1}^{J} \sum kD_{j,k}\tilde{G}\left( k-2n \right)$ | (3) |
| --- | --- | --- |

where $\hat{H}$ and $\hat{G}$ are low-pass filter and high-pass filter respectively, constituting the synthesis filter.

5.2.3 Signal Smoothing. We used moving average to smooth signals and removed extreme data points falling the upper and bottom five percentiles across all the signal spectrum.

5.2.4 Adjusting Personal Physiological Baselines**.** To account for personal effects on detecting seizure events, we considered personal baseline as the medians of physiological signals from each person, and calculated the residue values by subtracting the medians from the input signals.

**5.3 Signal analysis module**

The signal analysis module has the following components: (1) general analysis to derive overall distributions of the input signals; (2) data analysis for motion detection for three-axis ACC signals; (3) data analysis for detecting abnormal HR/HRV signals.

5.3.1 The general analysis component. Statistical analyses were performed to summarize the overall statistical distribution of signals and variables. These statistic data included maximum, minimum, average, median, standard deviation, Q1 (25% quartile), Q3 (75% quartile), 10% percentile, 90% percentile and peak differences)

5.3.2 Motion detection from ACC signals. The magnitude of net acceleration was calculated over all three axes of the accelerometer as the following:

|  | $a=\sqrt{a_{x}^{2}+a_{y}^{2}+a_{z}^{2}}$ | (4) |
| --- | --- | --- |

Sliding windows of every 5 minutes with 80% overlap (4 minutes) were used to calculate the standard deviation σ of a given acceleration epoch.

|  | $\sigma=\sqrt{\frac{1}{N-1}\sum_{i=1}^{N} \left( a_{i}-\mu\right)^{2}}$ | (5) |
| --- | --- | --- |

Epochs with σ below 0.1g were considered as resting states, and were removed from further analysis. When five-minute sliding window was used allowing 80% overlapping, there were at most 5 identifiers in an epoch. We took the one with more identifier as the epoch identifier.

5.3.3 HRV analysis. The HR signals were collected for heart rate viability analysis. Epochs with σ below 0.1 g were automatically discarded from further analysis and treated as non-motor state and hence non-seizure events. Next, R-R interval signals (i.e. distance between consecutive heart beats) were calculated from the valid HR signal through the PAWO pipeline. Finally, R-R interval signal in the 5-minute epochs were used to calculate HRV-associated parameters with sliding windows of 5 minutes with 80% overlap (4 minutes). The HRV-associated parameters used in PAWO included the following: standard deviation of normal R-R intervals (SDNN), root mean squared differences of the standard deviation (RMSSD), adjacent normal R-R interval of difference between the standard deviation (SDSD), the number of pairs of successive NNs that differ by more than 50 ms (NN50), the proportion of NN50 divided by total number of NNs (pNN50) in time domain, very low frequency (VLF, 0.001-0.04Hz), low frequency (LF, 0.04-0.15 Hz), high frequency (HF, 0. 15-0.4 Hz), the ratio of low frequency power and high frequency power (LF/HF), total power (TP) in frequency domain. Normalization was conducted on these HRV-associated parameters against the device build-in smoothing algorithm. When 5 minutes sliding window is used with 80% overlapping, there are at most 5 groups of HRV parameters in an epoch. We take the mean value as the groups of HRV parameters.

**5.4 Multi-signal time alignment module**

Using statistical analysis and machine learning to detect seizure onsets is essentially a pattern recognition problem for input signals in the time domain. Analyzing multi-channel signals from different sensor types requires unification of their respective time scale, leveraging the time stamps in our UNIX system. We aligned the signals using sliding windows based on the following resolution:

1. For quality check of input signals, the length of each sliding window was one minute, and there was no overlap between any two windows.
2. For both motion detection and HR/HRV analysis, the length of each sliding window was every five minutes, allowing 80% overlap between adjacent windows.

## 6. Physiome and activity study

The correlation analyses and statistical analyses were conducted on physiological signals to measure physiological changes across childhood developmental stages.

Multiple Linear Regression (MLR) analysis was independently conducted on 4 physiological signals (HR, GSR, ACC magnitude and SDNN) by controlling for age, sex and the time of the day. The pre-processing was performed as follows. First, the time period in which the human subject had a recorded seizure event were excluded from the analysis. Subsequently, the signals were preprocessed in the PAWO pipelines to denoise; outliners were dropped, and data was normalized by established baselines. In each individual MLR model, the response variables were calculated as the mean of the physiological signals in both daytime and nighttime; and the explanatory variables were age, sex and the time of the day. The linear model was written as the following:

|  | $Y_{d}=W_{d}^{T}X Y_{n}=W_{n}^{T}X$ | (6) |
| --- | --- | --- |

Take HR signal as an example, $Y_{d}\in R^{n\times1}, n=66$ represent the mean HRs of 66 participate in daytime (8 a.m. to 8 p.m.);$Y_{n}\in R^{n\times1}, n=66$ represent the mean HRs in nighttime (9 p.m. to 7.a.m.). Whereas $X\in R^{n\times m}, n=66, m=3$ represented the 3 physiological dimensions (age, sex, the time of the day) of the 66 participants. The **Equation 6** could further aggregate into the following:

|  | $\left[ Y_{d} Y_{n} \right]=[W_{d}^{T} W_{n}^{T} ]\left[ X X \right]$ | (7) |
| --- | --- | --- |

The contribution of age, sex and time were explained via regression coefficients $[W_{d}^{T} W_{n}^{T} ]$; their statistically significances were shown in **Figure 2A**.

Further statistical analyses were conducted on each physiological signal (HR, GSR, ACC and SDNN) to calculate the distribution characteristics across development stages. Boxplots were used to demonstrate the statistical characteristics, and Wilcoxon rank-sum tests were used to estimate statistical significance. The results were shown in **Figure 2** and **Supplemental Figure S6**.

## 7. Principal Component Analysis (PCA) on developmental physiologies during childhood development.

PCA is an unsupervised dimensional reduction (DR) technique for mining potential data structure, defined as,

|  | $\max_{W} WX^{T}XW^{T} s.t. W^{T}W=I$ | (8) |
| --- | --- | --- |

In our study, we implemented PCA to seek clustering pattern of physiology data of the human subjects by mapping from high dimensional space to low visible space, as follows,

|  | $Y=W^{T}X$ | (9) |
| --- | --- | --- |

Here, $X\in R^{n\times m}, n=66, m=70$, corresponding to 10 statistical features of 7 variables (HR, SDNN, 3-axis ACC, ACC magnitude, GSR) from 66 participants. $d$ was set as 3 in $X\in R^{n\times m}$ .

The raw signals from each sensor were processed through PAWO pipeline. SDNN variables and ACC magnitude were also calculated. Then, statistical analyses were conducted on these 7 groups of variables to acquire $66\times70 \left( n\times m \right)$ size matrix. Next, before implementing PCA algorithm, zero-mean standardization was applied to normalize features into scale $\left[ 0-1 \right]$. Finally, since PCA is an unsupervised method, the algorithm would output 66 three-dimensional $66\times3 \left( n\times d \right)$ samples in **Equation 9** without labeling. The age groups and gender were labeled to highlight the pattern of clustering. The results were shown in **Figure 3**, which indicated clear clustering pattern on age groups while little clustering by gender.

## 8. Construct prediction model for seizure onset

The overall prediction process was shown in **Supplemental Figure S2**. Multiple signals and variables were pre-processed and aligned in PAWO, including 3-axis ACC, 3-axis GYR, HR, GSR, ACC magnitude, GYR magnitude and R-R interval variables. Sliding window technique was applied to condense the data. Personal information and seizure onset times were also added to the window. Statistical procedures and HRV analysis were applied to extract relevant features. A bootstrapping-based ensemble network approach was used to recognize seizure onsets. The problem of seizure detection was posed as a supervised learning task, in which the goal was to classify each 60-second epoch as seizure or non-seizure based on extracted features from EDA and ACM recordings. If any epoch between the start and end of a labeled seizure was correctly classified as a seizure event, the seizure was considered detected (true positive). If multiple epochs within the seizure duration were detected, these were treated as a single true positive. False positives that occurred within 3 minutes from each other were treated as a single false alarm. Details are provided below.

Firstly, sliding window method (60 seconds long) was used to condense the time series into slices. The time window length was set as 60 seconds and overlapping set as 50%, i.e. 30 seconds. The time windows with more than 60% data missing were excluded. Then, according to the seizure events recorded by the nurse or caregivers, the time windows corresponding to seizure onsets were labeled as positive, the remaining windows were labeled as negative. In addition, 60 seconds buffer zones were added to the seizure events; these buffer zones were excluded as negative windows.

Next, features were extracted from each positive or negative window. These features included personal information such as age and sex, time series statistics and HRV variables. The time series parameters included heart rate, R-R interval, 3-axis accelerometer, accelerometer magnitude, 3-axis gyroscope, gyroscope magnitude and galvanic skin response. All the time domain and frequency domain HRV variables were calculated though PAWO. In summary, a total of 120 features from each 60 seconds window was calculated for the subsequent machine learning step to recognize seizure onset. These features are listed as follows:

1. Heart rate statistics (10): Maximum, Minimum, Mean, Median, Standard deviation, Q1 (25% Quartile), Q3 (75% Quartile), 10 percentile point, 90 percentile point and Peak value difference.
2. R-R Interval statistics (10): Maximum, Minimum, Mean, Median, Standard deviation, Q1 (25% Quartile), Q3 (75% Quartile), 10 percentile point, 90 percentile point and Peak value difference.
3. HRV variables (10): (SDNN), (RMSSD), (SDSD), (NN50), (pNN50) (VLF) (LF), (HF), (LF/HF), (TP)
4. GSR statistics (10): Maximum, Minimum, Mean, Median, Standard deviation, Q1 (25% Quartile), Q3 (75% Quartile), 10 percentile point, 90 percentile point and Peak value difference.
5. Accelerations and the magnitude statistics (40): Maximum, Minimum, Mean, Median, Standard deviation, Q1 (25% Quartile), Q3 (75% Quartile), 10 percentile point, 90 percentile point and Peak value difference.
6. Gyroscope and the magnitude statistics (40): Maximum, Minimum, Mean, Median, Standard deviation, Q1 (25% Quartile), Q3 (75% Quartile), 10 percentile point, 90 percentile point and Peak value difference.

Lastly, a bootstrapping-based embedding network approach, XGBoost (Chen et al 2016), was adopted from the sci-learn package (Pedregosa et al 2011) and used to overcome the imbalance between negative and positive groups. This model balances the distribution of positive and negative samples by bootstrap sampling and further alleviates the sample bias by embedding independent classifiers. The steps of algorithm are as follows,

1. Construct $n$ groups of bootstrap samples (denoted by $S_{i,i=1,2,\ldots n}$) from the original imbalanced population $\boldsymbol{S}$.
2. Balance sampling (sample equal number as positive) positive and negative in each group $S_{i,i=1,2,\ldots n}$. This practice yields a balanced dataset $D_{i,i=1,2,\ldots n}$
3. Construct stand-alone XGboost classifiers $X_{i,i=1,2,\ldots n}$ for each dataset $D_{i,i=1,2,\ldots n}$ as a branch of network.
4. Gather all classifier $X_{i,i=1,2,\ldots n}$ to generate an ensemble network $\left\{ X_{i} \right\}_{n=1,2,..,n}$.

The XGboost classifiers in each branch had equal effect. Therefore, the ﬁnal prediction score of bootstrapping-based ensemble network was averaged over the prediction scores output by all classifiers, i.e.,

|  | $Score\left( s \right)=\frac{1}{n}\sum_{i=1}^{n} X_{i}\left( s \right)$ | (10) |
| --- | --- | --- |

where, $n$ is the branch number of ensemble network.

A total of 734,933,434 data points collected from the discovery cohort were used to construct the XGboost model. The dataset was divided into 10-fold for cross-validation; leave-one-seizure-patient-out cross-validation was used. In the whole data set, the patients were divided into ten groups, and the total number of seizure onset was distributed as close as possible among the groups. In each validation process, nine groups were used as the training set, and the remaining one was used as the test set. In the training process, 64 branches were constructed in the bootstrapping-based ensemble network $\left\{ X_{i} \right\}_{n=1,2,..,64}$. XGboost classifiers in each branch shared the same parameter settings, which were tuned by the random grid-search technique. The configuration parameters were set as the following: number of estimators were set as 400, learning rate was set as 0.1, scale positive weight was set as 1, silent set was set as 0, subsample set was set as 0.9, regulation coefficients were set as 2 and 0, minimum child weight was set as 3, maximum tree depth was set as 8. The testing process was implemented on the trained XGBoost ensemble network.

Area Under Receiver Operating Characteristic curve (AUROC) was used to evaluate our model. The ROC curve was shown in **Figure 5A**, the contribution of features was shown in **Supplemental Figure S3**.

A critical threshold was selected to deem whether an outlier would be considered as an alarm and sent to the mobile end. This optimal decision threshold was selected at maximal slope at the tangent line of the ROC curve. In this study, the decision threshold was set as 0.5 in the model.

## 9. Construction of the cloud-based prediction system leveraging the pre-trained model.

An alarm system based on cloud computing was implemented using the trained XGBoost ensemble network with the optimized decision-making threshold. The alarm system possessed the complete training ensemble network $\left\{ S_{i} \right\}_{i=1,2,\ldots,64}$ from the blind validation procedure. All the samples were subsequently tested on our real-time data. The real-time data steam was continuously transmitted from wearable sensors to the backend cloud. In the cloud sever, PAWO executed the prediction algorithm every thirty second to analyze input signal at a time interval of 60 seconds. With the pre-trained model, we can immediately compute the prediction scores. When prediction scores were beyond our optimized threshold, the system immediately sent out warning messages to caregivers. Fast data transmission, cloud synchronization and automatic prediction has enabled fast decision making almost right after any abnormal physiological signals captured by the machine.

The alarm system was applied on 33 independently recruited children (whom were not involved in training the model). A total of 297,651,517 wearable sensor data points from these individuals were screened by our cloud system, and 11 out of the 33 children were recorded at least one seizure event. We compared the systems warning signal against clinical observations as well as against EEG signals whenever possible.

**References**

Chen T, Guestrin C (2016) XGBoost: A Scalable Tree Boosting System. KDD '16: Proceedings of the 22nd ACM SIGKDD International Conference on Knowledge Discovery and Data Mining.

Pedregosa F, Varoquaux G, Gramfort A, Michel V, Thirion B, et al. (2011) Scikit-learn: Machine Learning in Python. Journal of Machine Learning Research 12: 2825−2830.

Poh MZ, Loddenkemper T, Reinsberger C, Swenson NC, Goyal S, et al. (2012) Convulsive seizure detection using a wrist-worn electrodermal activity and accelerometry biosensor. Epilepsia 53: e93-97.
