# Supplementary figures and images for: Longitudinally tracking personal physiomes for precision management of childhood epilepsy
Source: PLOS Digit Health. 2022 Dec 19;1(12):e0000161. doi: 10.1371/journal.pdig.0000161 (PMC9931296; doi:10.1371/journal.pdig.0000161)

**A**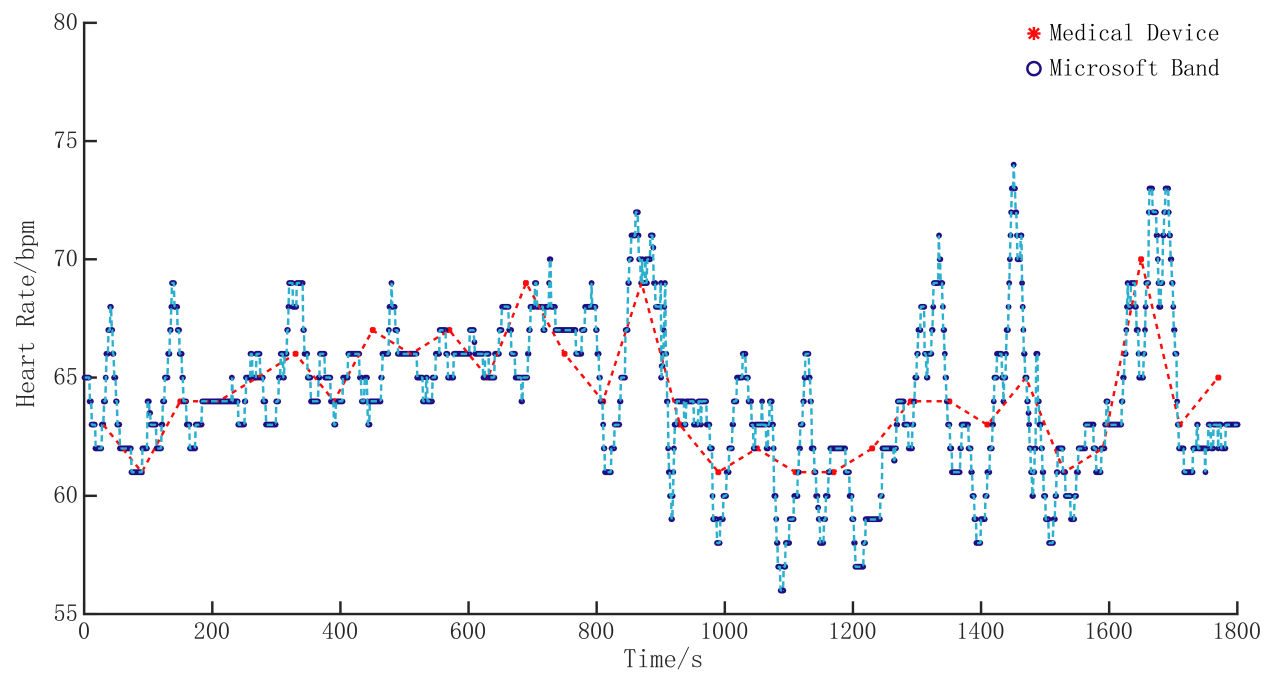**B**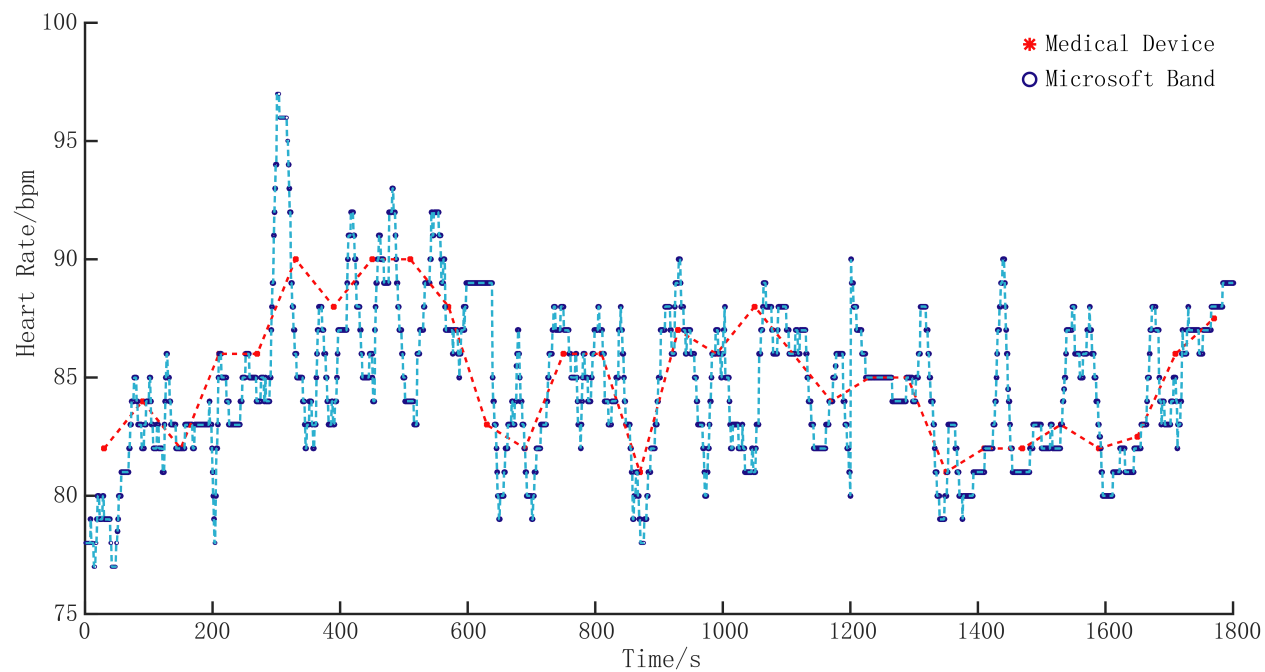**C**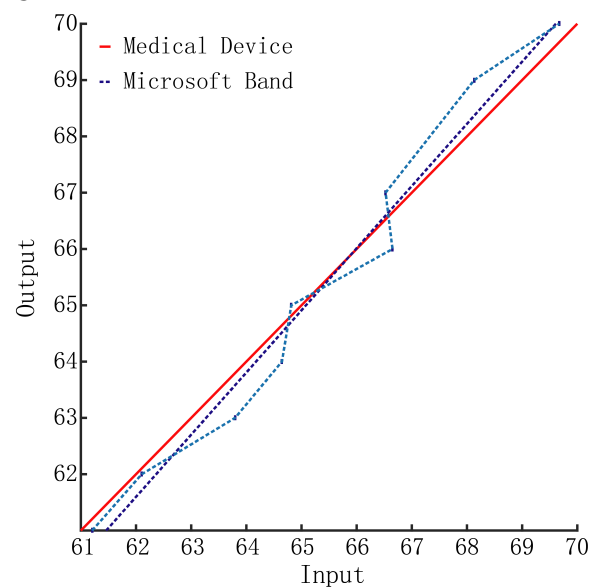**D**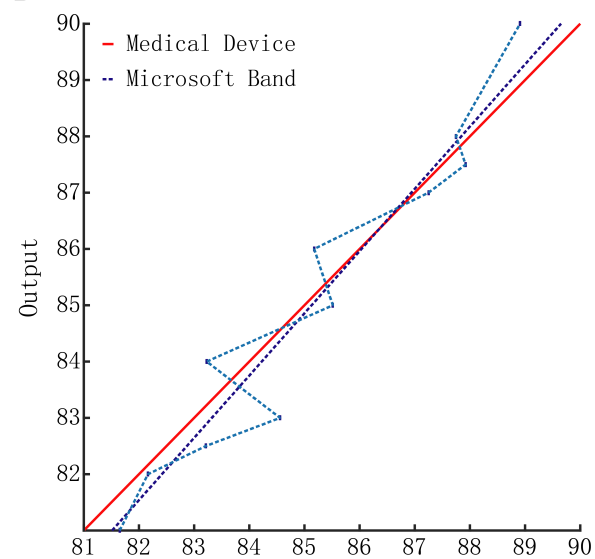

Supplement: S1 Fig — Benchmarking the HR measurements from the smart-band (Microsoft Band 1 and 2) and a medical grade device (OMRON HEM-6230) in the resting state (A) and in the active state (B). (C) The linearity analysis in the resting state, (D) The linearity analysis in the active state. (PDF) [file pdig.0000161.s003.pdf]

**A**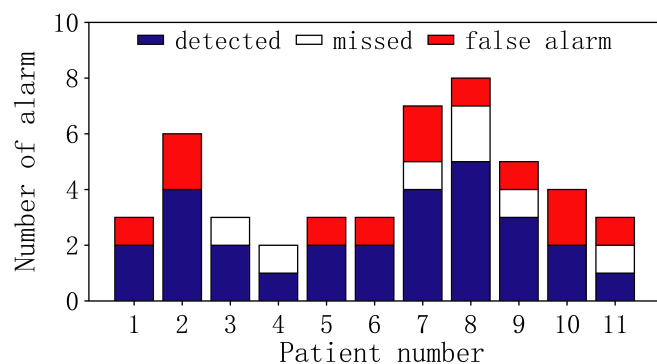**B**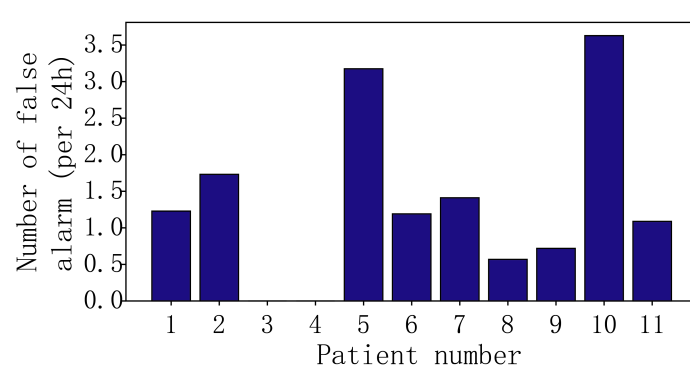**C**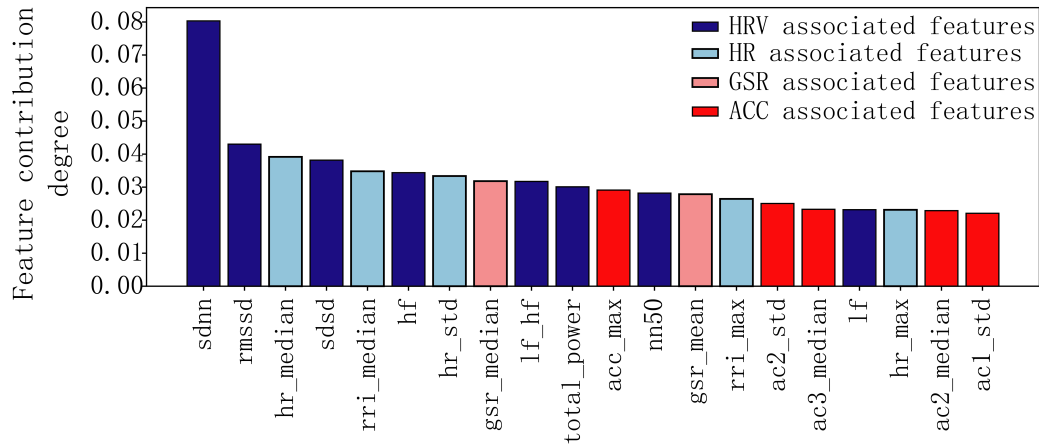

Supplement: S3 Fig — (A) Predicted seizure events from the 11 tested patients. Blue bars represent true positive seizure events as validated by the human observations, white bars represent seizure events recorded by human but missed by predictions, red bars represent false alarms, i.e., predicted seizure events but not observed by humans. (B) The number of false alarms per 24 hours on the tested 11 patients. (C) Estimated importance of the features in our seizure prediction model, in descending order of top 20, were SDNN, rMSSD, median of HRs, SDSD, median of R-R interval, HF, standard deviation of HRs, median of GSR, LF/HF, TP, maximum of ACC magnitude, NN50, mean of GSR, maximum of R-R interval, standard deviation of ACC in y axis, median of ACC in z axis, LF, maximum of HRs, median of ACC in y axis and standard deviation of ACC in x axis. (PDF) [file pdig.0000161.s005.pdf]

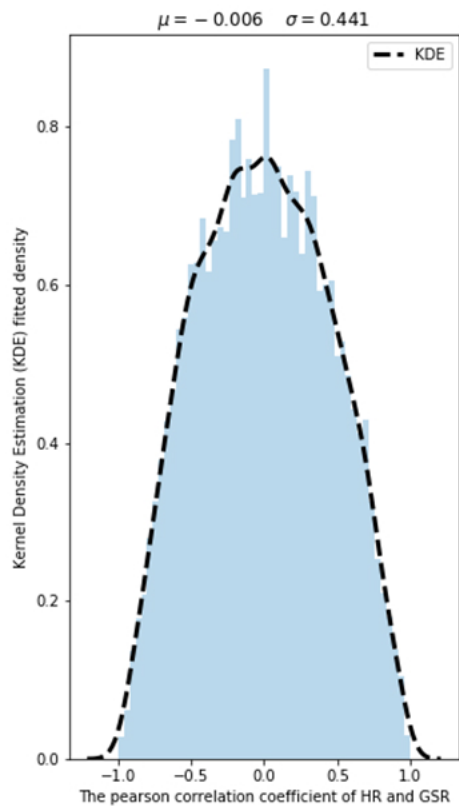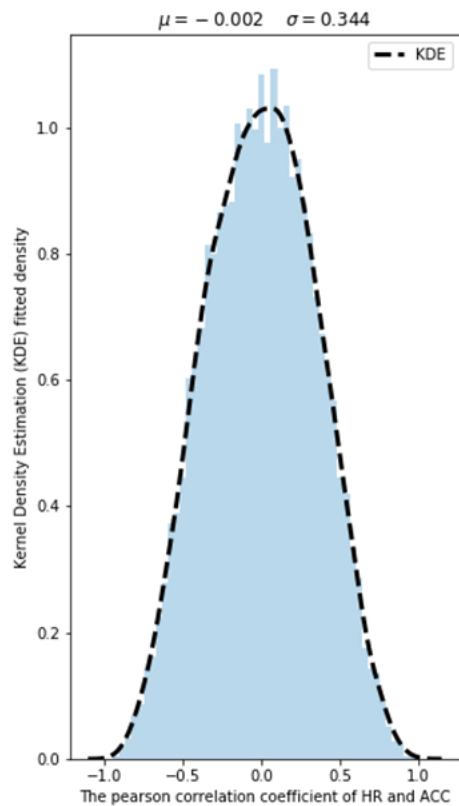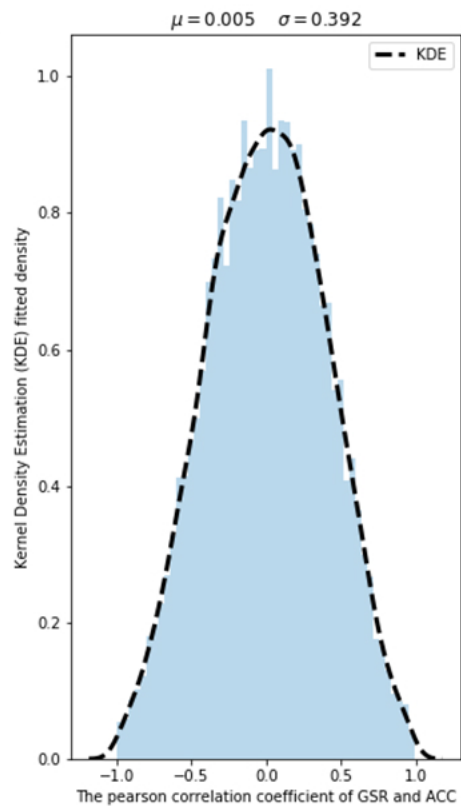

Supplement: S4 Fig — The plots show that there is minimal correlation between HR, ACC, GSR. (PDF) [file pdig.0000161.s006.pdf]

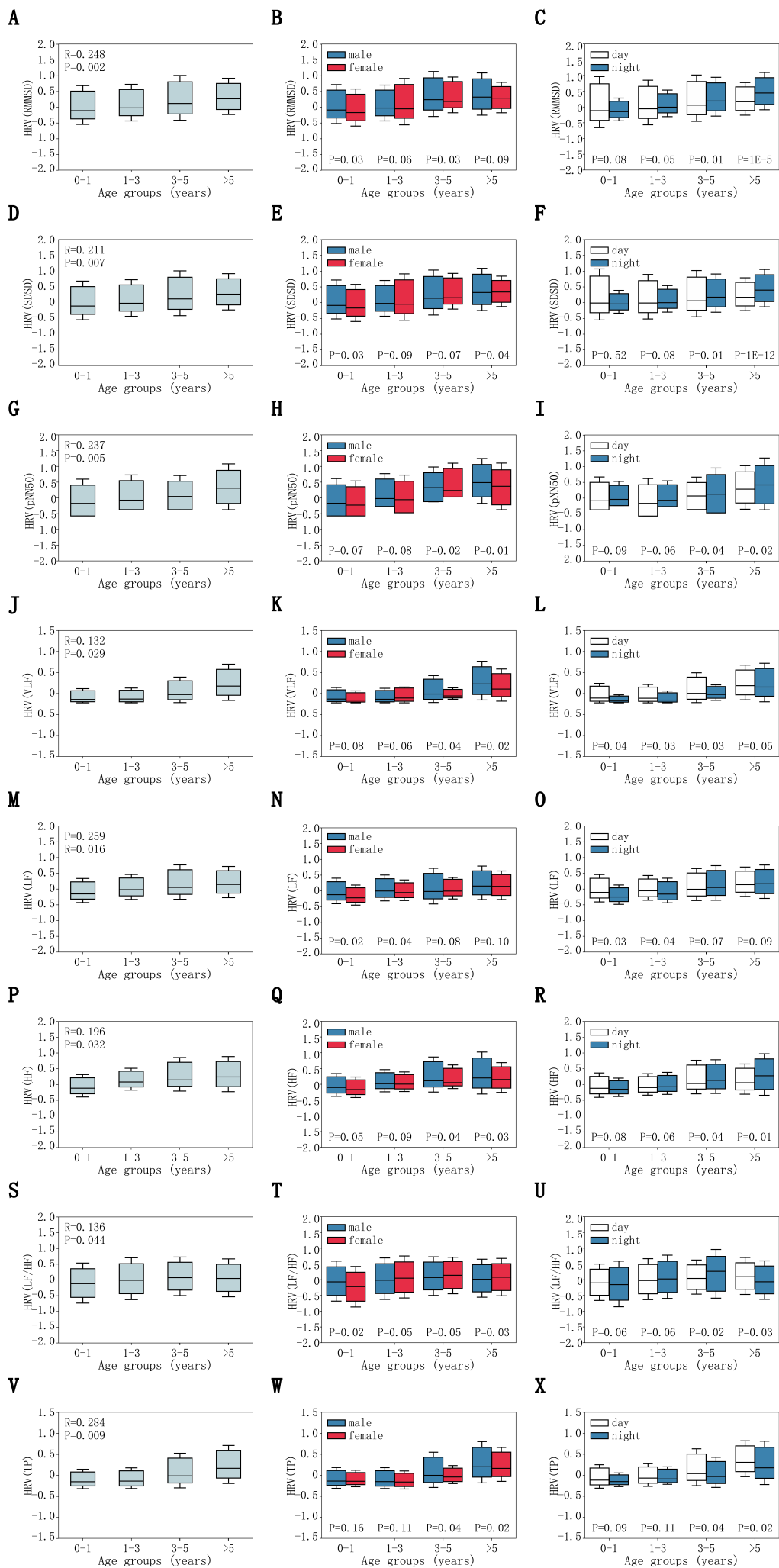

Supplement: S5 Fig — (A)-(I): time domain HRV associated parameters, (J)-(X): frequency domain HRV associated parameter. (A) rMSSD controlling for age. (B) rMSSD controlling for sex. (C) rMSSD controlling for the time of the day. (D) SDSD controlling for age. (E) SDSD controlling for sex. (F) SDSD controlling for the time of the day. (G) pNN50 controlling for age. (H) pNN50 controlling for sex. (I) pNN50 controlling for the time of the day. (J.) VLF controlling for age. (K) VLF controlling for sex. (L) VLF controlling for the time of the day. (M) LF controlling for age. (N) LF controlling for sex. (O) LF controlling for the time of the day. (P) HF controlling for age. (Q) HF controlling for sex. (R) HF controlling for the time of the day. (S) LF/HF controlling for age. (T) LF/HF controlling for sex. (U) LF/HF controlling for the time of the day. (V) TP controlling for age. (W) TP controlling for sex. (X) TP controlling for the time of the day. (PDF) [file pdig.0000161.s007.pdf]
