# Supplementary material for: Longitudinally tracking personal physiomes for precision management of childhood epilepsy
Source: PLOS Digit Health. 2022 Dec 19;1(12):e0000161. doi: 10.1371/journal.pdig.0000161 (PMC9931296; doi:10.1371/journal.pdig.0000161)
Supplement: S2 Fig — The pipeline consists of the following sequential functional modules: Pre-processing, Segmentation, Feature Extractions and Classification. (PDF) [file pdig.0000161.s004.pdf]

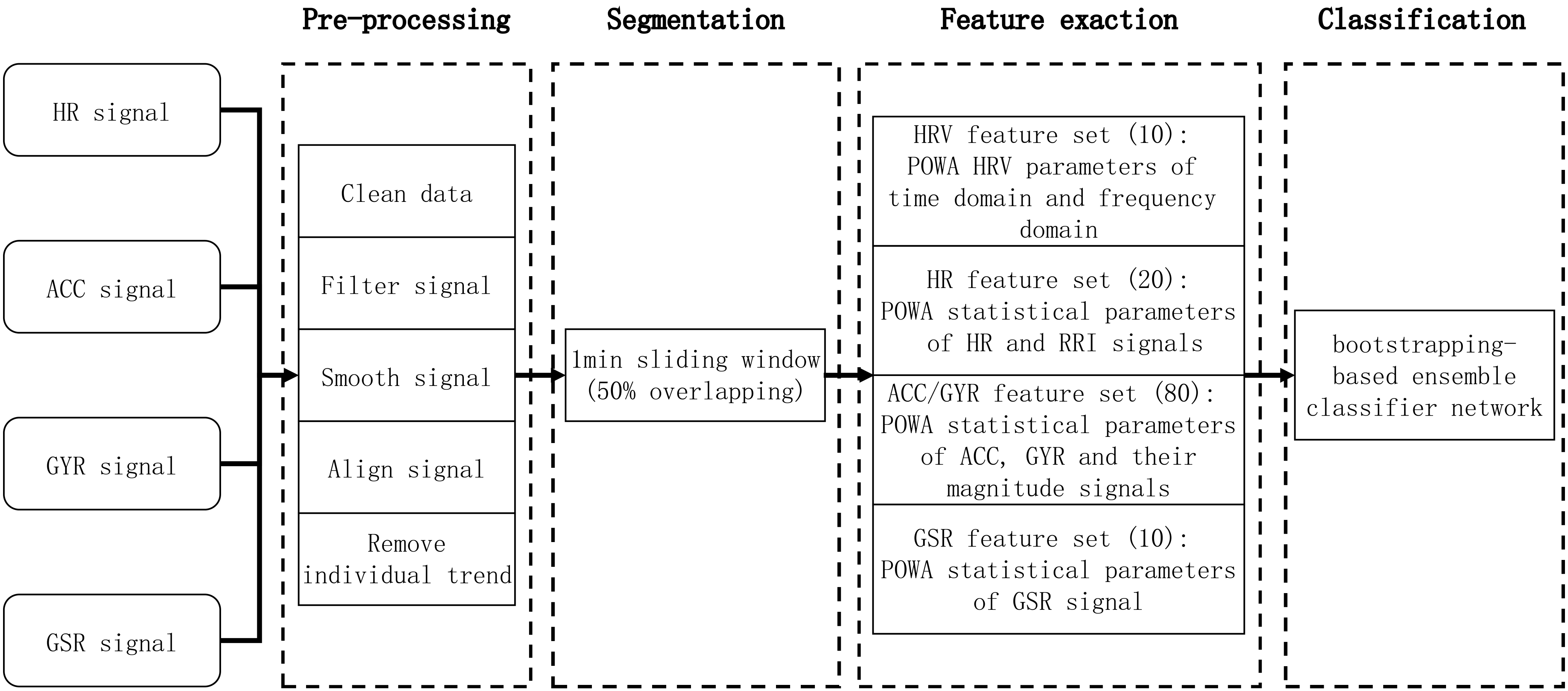
Pre-processing
Segmentation
Feature exaction
Classification
HR signal
ACC signal
GYR signal
GSR signal
Clean data
Filter signal
Smooth signal
Align signal
Remove individual trend
1min sliding window (50% overlapping)
HRV feature set (10): POWA HRV parameters of time domain and frequency domain
HR feature set (20): POWA statistical parameters of HR and RRI signals
ACC/GYR feature set (80): POWA statistical parameters of ACC, GYR and their magnitude signals
GSR feature set (10): POWA statistical parameters of GSR signal
bootstrapping-based ensemble classifier network
